# LMO1 Plays an Oncogenic Role in Human Glioma Associated With NF-kB Pathway

**DOI:** 10.3389/fonc.2022.770299

**Published:** 2022-02-24

**Authors:** Lei Gao, Jia Wu, Hai Wang, Yongyu Yang, Zongliao Zheng, Bowen Ni, Xiran Wang, Yuping Peng, Yaomin Li

**Affiliations:** ^1^ Department of Neurosurgery, Nanfang Hospital, Southern Medical University, Guangzhou, China; ^2^ Laboratory for Precision Neurosurgery, Nanfang Hospital, Southern Medical University, Guangzhou, China; ^3^ Department of Hepatobiliary Surgery, Nanfang Hospital, Southern Medical University, Guangzhou, China

**Keywords:** LMO1, NGFR/p75 NTR, NF-kB pathway, glioma, bioinformatics analysis

## Abstract

**Background:**

LIM domain only protein1(LMO1), a nuclear transcription coregulator, is implicated in the pathogenesis of T-cell acute lymphoblastic leukemia and neuroblastoma. However, the clinical significance and potential mechanism of LMO1 in human gliomas remain to be determined.

**Methods:**

In this study, expression level data and clinical information were obtained *via* three databases. The Cox proportional hazards regression model was used to predict outcomes for glioma patients. *In vitro* and *in vivo* assays were used to explore the function of LMO1 in human glioma. Gene set enrichment analysis (GSEA), RNA-seq and western blot were used to explore the potential molecular mechanisms. A prognostic model was built for predicting the overall survival(OS) of human glioma patients.

**Results:**

High LMO1 expression was associated with a high tumor grade and a poor prognosis in patients. High levels of LMO1 mRNA were correlated with poor prognosis in patients with isocitrate dehydrogenase (IDH)-wild-type (wt) and 1p/19q non-codeletion gliomas. Gene silencing of LMO1 significantly inhibited tumor growth, invasion and migration *in vitro*. In contrast, LMO1 over-expression promoted tumor growth, invasion and migration. Mechanically, LMO1 may positively regulate the level of NGFR mRNA and protein. NGFR mediated the regulation between LMO1 and NF-kB activation. Consistently, the nude mice study further confirmed that knockdown of LMO1 blocked tumor growth *via* NGFR-NF-kB axis. Finally, The nomogram based on the LMO1 signature for overall survival (OS) prediction in human glioma patients exhibited good performance in the individual mortality risk.

**Conclusion:**

This study provides new insights and evidences that high level expression of LMO1 is significantly correlated with progression and prognosis in human gliomas. LMO1 played a critical role in tumorigenesis and progression. The present study first investigated the LMO1–NGFR–NF-kB axis regulate cell growth and invasion in human glioma cells, whereby targeting this pathway may be a therapeutic target for glioma.

## Introduction

Human glioma is the most prevalent primary tumors of the brain, and it has an aggressive malignant progression represented by devastation to normal brain tissue, resistance to therapeutic approaches, and widespread invasion throughout the brain (1, 2). The past several decades of dedicated research into the biology of glioma has resulted in a rapidly accelerating process of discovery. Although many molecular signatures have been reported to be closely related with fundamental characters of glioma, the clinical treatment effect of glioma is still unsatisfactory. Therefore, elucidating the key mechanism controlling glioma cell growth and invasion and searching therapies is essential for improving patient survival (3). Various public databases have collected abundant genetic and clinical information of glioma, which is helpful to screen out new therapeutic targets and improve clinical prognosis for patients.

The LIM-only (LMO) proteins are a family of nuclear transcription coregulators, which are characterized by the exclusive presence of two tandem LIM domains and no other functional domains. The LIM domain is an ~55-residue, highly conserved cysteine-rich zinc-binding motif (5, 6). To date, four LMO proteins (LMO1-LMO4) have been identified. LMO proteins are emerging as crucial molecules in a wide variety of human cancers. LMO1, a member of LMO family, is reported as a dominant oncogene in neuroblastoma cells (7). Silence of LMO1 may suppress the growth of neuroblastoma cells with high LMO1 expression, whereas overexpression of LMO1 in neuroblastoma cells with low LMO1 expression promotes proliferation (7).

Nerve growth factor receptor, known formally as “Protein tumor necrosis factor receptor superfamily member 16” and also called p75 neurotrophin receptor (NGFR), is a transmembrane glycosylated receptor that elicits an array of biological functions through its ability to interact with its cognate ligands and coreceptors (8–10). It is well-known that NGFR alone or with other coreceptors can mediate several cellular functions that include cell death, survival, migration, and axonal growth inhibition (11–13). In glioma, p75NTR can modulate hallmarks of glioblastoma including invasion and proliferation (14–18).

Our previous studies have revealed that NF-κB activation promotes the glioblastoma mesenchymal phenotype (19). In cells, IκB interacts with NF-κB (p65), leading to NF-κB (p65)/IκB complex sequestration in the cytoplasm, and subsequent prevention of NF-κB(p65) binding to target DNA sequences. Some signal cascades activate IKK, and IKK phosphorylates IκB in the cytoplasm, resulting in IκB degradation by the proteasome and NF-κB (p65) release from the inhibitory complex. Then, NF-κB proteins translocate into the nucleus, where they bind to DNA and activate gene transcription (20). The pathway plays an important role in cancer cell proliferation, apoptosis, metastasis, and therapeutic resistance.

This is the first report of LMO1 as a prognostic predictor and its function in human glioma cells. We found that LMO1 expression is significantly increased in more aggressive gliomas. LMO1 was revealed as a transcriptional cofactor, affecting the NF-κB pathway by regulating the NGFR expression. Our data suggested that LMO1, as a novel biomarker of gliomas, plays an important role in gliomas though the NF-κB signaling pathway.

## Materials And Methods

### Clinical Specimens and Databases

Gene expression and glioma patient survival data were downloaded from The Cancer Genome Atlas (TCGA) Research Network (n = 676), The Chinese Glioma Genome Atlas (CGGA) database (n = 693), and the GSE16011 (GEO) database (n = 284). RNA-seq data of 301 glioma patients with clinicopathologic characteristics from the CGGA were selected as the primary cohort to establish the predictive model and to construct the nomogram and risk classification system. The inclusion criteria for data extraction in the predictive model were patients diagnosed with WHO grade II–IV glioma. The exclusion criteria included patients with missing or incomplete data such as survival status and time, age, sex, grade, and IDH status. Archived paraffin embedded glioma tissues (WHO grades I–IV) were gathered from patients (n = 37) who underwent surgery in the Department of Neurosurgery, Nanfang Hospital of Southern Medical University. Normal brain tissue samples (n = 5) were collected from severe traumatic brain injury patients who experienced partial resection of the normal brain as decompression treatment.

### Patient-Derived Glioma Specimens and Cell Lines

All glioma samples were obtained after surgical resection from patients admitted to the Department of Neurosurgery, Nanfang Hospital (NFH), Southern Medical University, China, and the corresponding clinical data were collected. The glioma specimens were obtained for pathological examination and cell isolation. The primary cell lines, NFH-GBM1 (derived from a 61 year-old male GBM patient), were isolated from fresh GBM tissues. The maintained adherent cells were primary GBM cells, which were verified by immunofluorescence,as previously described (19). The canonical GBM cell lines (LN229, U87MG, T98G and U251) and normal glial cell lines (SVG)were purchased from the American Type Culture Collection (ATCC, USA). The cell lines were cultured in Dulbecco’s modified Eagle’s medium (DMEM; Gibco; USA) that was supplemented with 10% foetal bovine serum (FBS; Gibco; USA). The details of the primary GBM cell lines were listed in **Supplementary Table S1**.

### Immunohistochemistry (IHC)

Immunohistochemistry assays were carried out on GBM samples or nude mouse xenograft tumour tissues to detect and score LMO1(ab137599, Abcam), p-p65 (ab86299, Abcam), NGFR(55014-1-AP, Proteintech), anti-Ki67 (ZM-0166, ZSGB-BIO) and Vimentin(#9782, Cell Signaling Technology)expression. Paraffin-embedded blocks were cut into 4-μm sections and deparaffinized and rehydrated. Antigen retrieval was performed by pressure cooking for 5 min in citrate buffer (pH 6.0), followed by blocking of endogenous peroxidase in 0.3% H2O2. After blocking with 5% bovine serum albumin (BSA) for 1 h, sections were incubated sequentially with primary antibodies and horseradish peroxidase-linked secondary antibody. Sections were covered with diaminobenzidine for visualizing the staining and then counterstained with haematoxylin before being examined by microscope. The staining intensities were recorded as 0 (negative), 1 (weak), 2 (moderate), and 3 (strong), and the percentage of positively stained cells was recorded as 0 (0-25%), 1 (25-50%), 2 (50-75%), and 3 (75-100%). IHC scores were obtained by multiplying the two abovementioned scores. The median score, which was 6, was regarded as the cutoff for distinguishing “high expression” and “low expression” of LMO1.

### Immunofluorescence (IF)

For immunofluorescence, 104 cells were grown on 15 mm confocal petri dishes and transfected with lentiviral for the indicated time. Cells were fixed for 10 min in 4% paraformaldehyde, permeabilized with 0.5% Triton™ X for 10 min and blocked with 5% BSA for 1 h. After removal of BSA, cells were rinsed with PBS and incubated with anti-p65 antibody (#6956S, Cell Signaling Technology) at 37°C for 1h. Immunofluorescence staining was enhanced using Alexa Fluor^®^ 488-labelled secondary antibodies (Invitrogen). Cells were also stained with DAPI. Images were captured under a Carl-Zeiss LSM 980 confocal microscope equipped with ZEN 3 software (version 4.0) for image acquisition and analysis.

### Gene Set Enrichment Analysis (GSEA)

To gain insight into the biological processes and signal pathways associated with LMO1 expression in gliomas, GSEA was performed using the Broad Institute GSEA version 4.0 software. The CCGA database was downloaded. The gene sets used for the enrichment analysis were downloaded from the Molecular Signatures Database (MsigDB, http://software.broadinstitute.org/gsea/index.jsp).

### RNA Extraction and qPCR

mRNA levels of LMO1 were detected by qPCR. Total RNA was extracted using TRIzol Reagent (Invitrogen) according to the manufacturer’s protocol. Total purified RNA was reverse transcribed with random primers using cDNA synthesis kit (Thermo Fisher Scientific, Rockford, IL, USA). By the resulting cDNA used as a template for RT-PCR, real-time PCR using SYBR Green (Thermo Fisher Scientific, Rockford, IL, USA) was performed to detect the mRNA levels of the indicated genes. GAPDH was served as an internal control. The following primers were synthesized by Invitrogen: (LMO1 forward: 5’-TGGAAATCAAGAAACAGATG GA -3’; reverse:5’-TGGAGATGGGGCTCAGGTA-3; NGFR forward: 5’-CCTGGACAGCGTGACGTTC -3’, NGFR reverse:5’-CCCAGTCGTCTCATCCTGGT-3). All reactions were conducted on an ABI 7300 real-time PCR machine (Applied Biosystems, Foster City, CA, USA) using the following cycling parameters: 95°C for 10 min, followed by 40 cycles of 95° C for 15 s and 60°C for 45 s. Quantitation of relative gene expression was calculated using comparative△△Ct method. All data represent the average of three replicates.

### Cell Transfection

Small interfering RNA (siRNA) targeting LMO1 were synthesized (Umine Biotechnology; Guangzhou, China). siRNAs were transfected with Lipofectamine™ 2000 reagent (Thermo Fisher Scientific; USA) according to the manufacturer’s protocol. Knockdown efficiency was assessed 72 h after transfection by western blotting. Stable knockdown of LMO1 in cells was generated using lentiviral transduction of shLMO1 (Hanbio BiotechnologyCoLtd; Shanghai, China). Knockdown efficiency was assessed 72 h after transfection by western blotting. siRNA sequences that generated efficient knockdown are as follows: si-LMO1sense:5′-CGCGACUACCUGAGGCUCUUUdTdT-3′; antisense:AAAGAGCCUCAGGUA GUCGCGdTdT. Plasmid construction of pIRES2-LMO1 and pIRES2-NGFR were performed by Umine Biotechnology (Guangzhou, China).

### Western Blotting

Harvested cells were lysed with heat denaturation in RIPA cell lysis buffer. Protein lysates (20 μg) were loaded and separated on SDS-PAGE, and the proteins were transferred to polyvinylidene difluoride (PVDF) membranes. The blots were incubated with primary antibodies against LMO1(ab137599, Abcam),NGFR(55014-1-AP, proteintech),MMP2(#40994,Cell Signaling Technology), NF-κB Pathway Sampler Kit antibodies (#9936, Cell Signaling Technology), Epithelial-Mesenchymal Transition (EMT) Antibody Sampler Kit antibodies (#9782, Cell Signaling Technology),GAPDH(AP0066-200,Bioworld); Specific proteins were visualized with enhanced chemiluminescence (ECL, Millipore, Bredford, USA). The intensity of the protein bands was measured (ImageJ software) and normalized to GAPDH.

### Cell Counting Kit (CCK)-8 Assay

The Cell Counting Kit-8 (CCK-8) was used to evaluate the cell viability according to the manufacturer’s instructions (Dojindo, Japan). NFH-GBM1, LN229 cells (1 × 103 cells/well) were incubated in 96-well plates for 0, 2, 4 and 6 d. The CCK-8 solution (10 μL) was added to each well and the plates were incubated for 1 h at 37°C, and then the absorbance at 450nm wavelength (OD450) was measured in a Microplate Reader (Bio-Rad).

### Cell Scratch Assays

All cell lines were cultured in 6-well plates. Cells were grown to 85% confluence, scratched in each well with a new 1 mL pipette tip, washed twice with PBS to remove the scraped cells, and treated with eupatilin for 24 and 48 hrs. Images were taken and the gap distance was quantified using Image J software.

### Transwell Invasion Assays

To further assess invasiveness, the filters were precoated with Matrigel. Glioma cells were added to the top chamber in serum-free media. The bottom chamber was filled with 10% FBS DMEM. After 24–48 h of incubation, the top chamber cells were removed using a cotton swab, and the membrane was fixed in 4% paraformaldehyde for 15 min and stained with crystal violet for 15 min. Five fields of adherent cells in each well were photographed randomly.

### RNA-Seq

Three biological replicates in each control and knockdown LMO1 groups in LN229 cells were collected for RNA-Seq analysis after 72h of transient transfection. Total RNA isolation, library construction and sequencing were conducted using an Illumina HiSeq 2000 system, following the standard instructions. The differentially expressed genes were screened based on fold change (>=1) and Student’s t-test (P <0.05).

### *In Vivo* Assays

To establish intracranial gliomas, LN229 cells (1 × 10^7)^ were transfected with Lenti-sh-LMO1 or LentiControl virus and then stereotactically implanted into the brains of 4-week-old nude mice (SMU Laboratory Animal Center; guangzhou, China). Kaplan-Meier survival curves were plotted to determine the survival time and weight. Mice were monitored until neurological signs were observed or 50 days after implantation, at which point they were sacrificed.

### Statistical Analysis

Statistical analyses were performed using GraphPad Prism 8 software. Student’s t tests, one-way ANOVA, Pearson correlation analyses, Kaplan-Meier analyses, log-rank tests, Cox’s proportional hazards regression model, and χ2 tests were used to analyse the corresponding data,as detailed in the figure legends. The nomogram and risk classification system were constructed using rms package and predict function respectively in R. The performance of the nomogram was measured by ROC curve and calibration curve established in R.

## Results

### LMO1 Is Upregulated in High-Grade Glioma Patients

**Figure 1:**
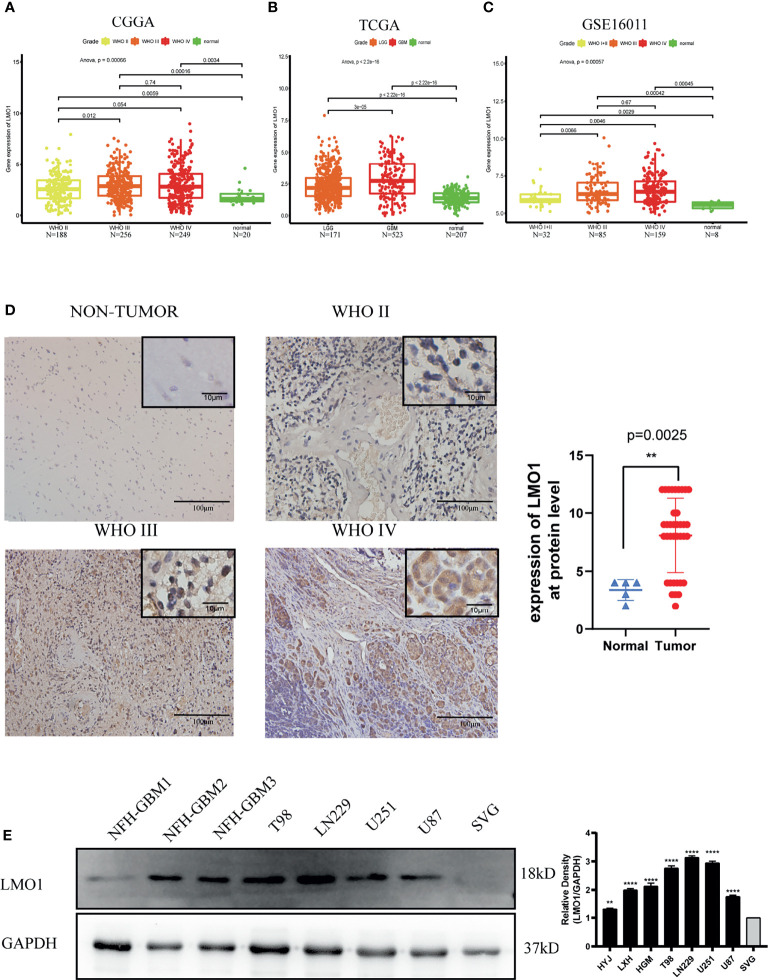
Increased LMO1 expression is related to a more malignant glioma. **(A)** The expression levels of LMO1 in 693 tumor tissues and 20 normal tissues were analyzed by CGGA. **(B)** The expression levels of LMO1 in 694 tumor tissues and 207 normal tissues were analyzed by TCGA. **(C)** The expression levels of LMO1 in 276 tumor tissues and 8 normal tissues were analyzed by GSE16011.Tukey’s Honest Significant Difference (HSD) test is used in **(A–D)** Representative immunohistochemistry (IHC) staining for LMO1 in non-tumor and different grade glioma samples. Scale bars=100 μm (main images) and 10 μm (insets).Comparison of LMO1 IHC staining scores between non-tumor and glioma samples. Unpaired Student’s t-test were used for statistical analysis. **(E)** WB analysis of LMO1 in the canonical and patient-derived GBM cell lines. GAPDH was used for normalization. One-way ANOVA tests were used for statistical analysis. **p < 0.01. ****p < 0.0001.

To understand the function of LMO1 in glioma development, we investigated the expression level of LMO1 in multiple datasets. A total of 693 tumor samples from glioma patients and 20 normal samples from CGGA databases were analyzed, The data indicated that expression level of LMO1 was higher in tumor samples than in control samples. Furthermore, the results further indicated that the mRNA expression levels of LMO1 were significantly increase with the rise in the grade of glioma and that the expression level was highest in the group of WHO IV (**Figure 1A**). Moreover, in the TGGA and GSE16011 databases, higher expression of levels of LMO1 were observed in higher grade glioma or GBM samples than those in low grade glioma and normal samples (**Figures 1B, C**). Correspondingly, the expression of the LMO1 protein was higher in human gliomas (WHO IV, n = 31;WHO II-III, n = 6; **Supplement Table 2**) than in normal brain tissues (n = 5) (**Figure 1D**). Clearly, LMO1 protein were mainly localized in the nuclear of glioma cells. These findings were further confirmed by western blot analysis, which investigated LMO1 expression in GBM cell lines (T98,LN229, U87 and U251 cell) and primary glioma cell lines (NFH-GBM1, NFH-GBM2, NFH-GBM3 cell) and normal glial cells (SVG cell) (**Figure 1E**). Thus, high LMO1 expression was correlated with an increased tumor grade in glioma patients.

### LMO1 Served as an Independent Prognostic Factor for Glioma Patients

**Figure 2:**
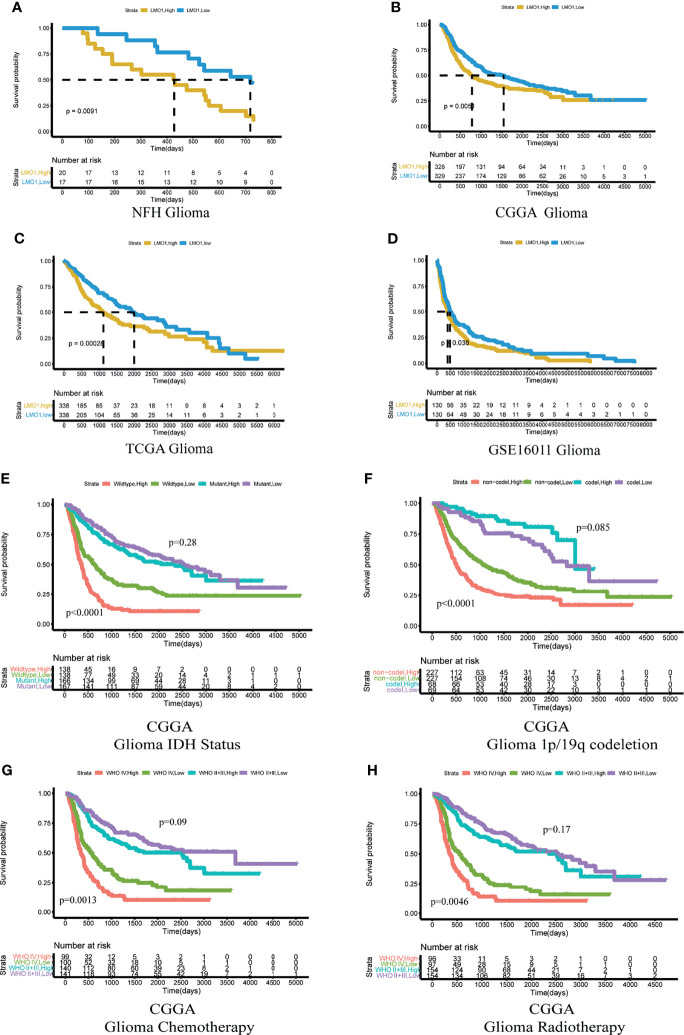
High expression of LMO1 predicts poor clinical outcomes in human gliomas and the response to chemo- and radio-therapy. **(A)** Survival curves including all glioma patients enrolled at NFH stratified by the IHC staining score for LMO1. an IHC staining score of 6 was regarded as the cutoff. The log-rank test was used to calculate the p-value. **(B–D)** Survival curves of glioma patients in the CGGA dataset **(B)**, TCGA dataset **(C)** and GSE16011 dataset **(D)** stratified by LMO1 expression. The median mRNA expression level was regarded as the cutoff between high and low LMO1 expression. **(E, F)** Kaplan–Meier analysis of the survival of glioma patients considering the mutation status of IDH(E)or 1p/19q codeletion status **(F)** from the CGGA according to LMO1 expression. **(G, H)** Kaplan–Meier analysis of the survival of glioma or GBM patients treated with chemotherapy **(G)** or radiotherapy **(H)** from the CGGA according to LMO1 expression. Log-rank tests were used to calculate p-values.

To investigate the further relationship between LMO1 expression and clinical prognosis, we collected survival data from 42 patients with different grades of glioma (**Supplementary Table S2**). The results demonstrated that elevated LMO1 expression was clinically correlated with unfavorable outcomes of glioma patients outcome (**Figure 2A**). To further confirm this result, we downloaded survival data from the TCGA, CGGA and GSE16011 databases and performed survival analysis to investigate the clinical relevance of LMO1 expression in patient survival. The results display that elevated LMO1 expression was clinically correlated with unfavorable outcomes of glioma patients (**Figures 2B–D**). The detection of several molecular markers, including IDH1 mutation, 1p/19q codeletion, MGMT promoter methylation status has been applied with clinical diagnoses of gliomas (21, 22), we analyzed whether LMO1 expression was correlated with some molecular genetic characteristics by using CGGA database. The data revealed that the expression of LMO1 mRNA in human gliomas was correlated with 1p/19q co-deletion and MGMT promoter methylation status but not IDH status (**Table 1**). Results of Kaplan-Meier survival analyses stratifified by the status of common genetic aberrations of glioma showed that the effect of LMO1 on patients’ prognosis was correlated with several molecular features including IDH methylation and 1p/19q non-codeletion. It was rather remarkable that the prognostic significance of LMO1 was highly pronounced in individuals with wild type IDH or 1p/19q non-codeletion (**Figures 2E, F**). The results also in the CGGA revealed that the glioblastoma patients receiving chemotherapy or radiotherapy in the high LMO1 expression group had a poorer prognosis than patients in the low LMO1 expression group (**Figures 2G, H**).

**Table 1:** Relationship between LMOl expression and clinicopathological Characteristics of GBM patients based on CGGA (n = 656).

Characteristics	LMOl		X^2^	P value
	Low expression (N)	High expression (N)		
Age(y)				
≤60	296	292	0.263	0.608
>60	32	36		
Gender				
male	204	169	7.613	0.006
female	124	159		
Grade				
WHOII	99	73	6.110	0.047
WHOIII	112	135		
WHOIV	117	120		
PRS Type				
primary	208	195	1.087	0.297
recurrent	120	133		
IDH Mutation				
mutant	170	163	2.263	0.323
wildtype	139	136		
unknown	19	29		
MGMT Methylation				
methylated	132	172	11.442	0.003
unmethylated	115	102		
unknown	81	5 1		
lpl9q Codeletion				
codel	62	75	45.561	<0.001
non-codel	207	246		
unknown	59	7		

Moreover, subsequent univariate and multivariate Cox regression analyses were additionally conducted to determine the independence of the prognostic value of LMO1. After correction for clinical characteristics that LMO1 were suggested to be significant prognostic factors in the univariate Cox regression, high LMO1 expression was an independent risk predictor of OS (p = 0.001, hazard ratio (HR) = 1.429, 95% confidence interval (CI) = 1.152–1.733) for glioma patients (**Table 2**). Collectively, these results indicate that LMO1 could be an independent prognostic factor for human glioma.

**Table 2:** Univariate and multivariate Cox regression analysis for OS in GBM patient based on CGGA (n = 542).

Variable	Univariate analysis			Multivariate analysis		
	HR	95% CI	P value	HR	95% CI	P value
LMO1	1.429	1.152-1.773	0.001	1.697	1.358-2.122	<0.001
Age	2.253	1.651-3.073	<0.001	1.153	0.834-1.593	0.388
Gender	0.912	0.734-1.133	0.405	0.925	0.741-1.154	0.489
Grade						
WHOII	reference			reference		
WHOIII	2.899	2.033-4.134	<0.001	2.623	1.826-3.769	<0.001
WHOIV	7.778	5.475-11.048	<0.001	4.794	3.296-6.974	<0.001
PRS Type	2.157	1.735-2.681	<0.001	2.180	1.5748-2.720	<0.001
IDH Mutation	3.063	2.458-3.817	<0.001	1.766	1.345-2.320	<0.001
1p19q Codeletion	3.572	2.545-5.015	<0.001	2.410	1.678-3.461	<0.001

### LMO1 Promotes Glioma Cell Proliferation, Migration and Invasion *In Vitro*

**Figure 3:**
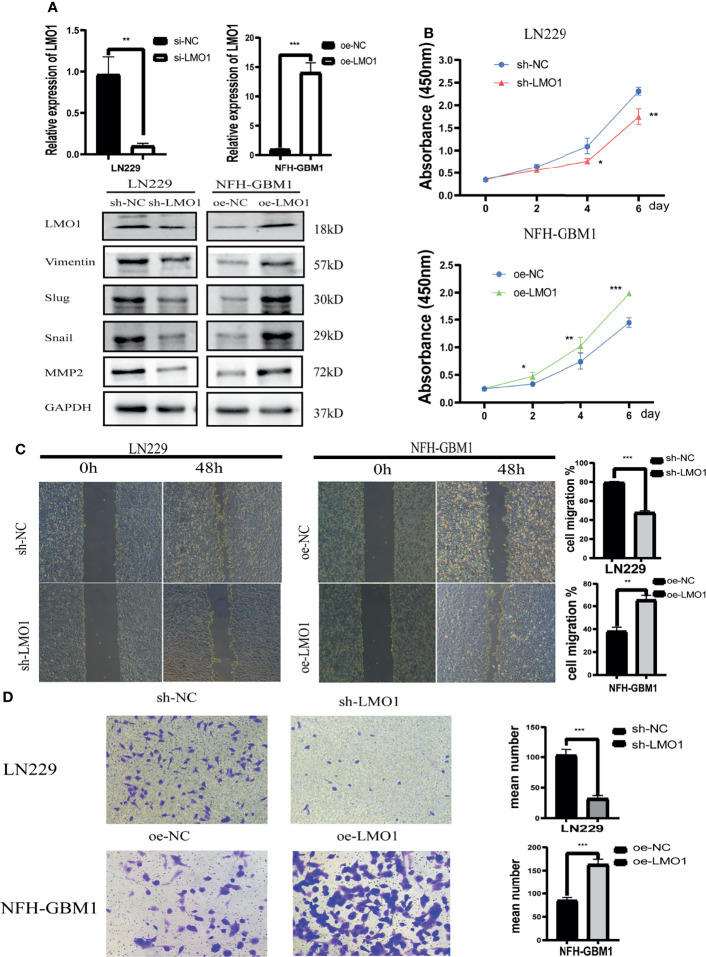
*In vitro* assays revealed that LMO1 is essential for glioma tumorigenesis and progression. **(A)** qPCR and WB analysis of LMO1 in LN229 cells or NFH-GBM1cells after the indicated shRNA or overexpression transfection. GAPDH was used for normalization. Unpaired Student’s t-tests was used for statistical analysis. **(B)** Cell counts of surviving LN229 cells and NFH-GBM1cells 6 days after the indicated transfection. Six technical replicates were performed for each group. One-way ANOVA was used for statistical analysis. **(C)** Scratch assays of LN229 cells and NFH-GBM1cellsafter transfection. At least five independent fields of cells were counted and measured. Three biological replicates were performed. Student’s t test was used for statistical analysis. **(D)** Transwell assays of LN229 cells and NFH-GBM1cell after transfection. Original magnification, 400×. Five random fields of view were captured for each group. Three biological replicates were performed(left panel). Quantification of transwell assay is shown in the right panel. Student’s t test was used for statistical analysis. *p < 0.05. **p < 0.01. ***p < 0.001.

**Figure 4:**
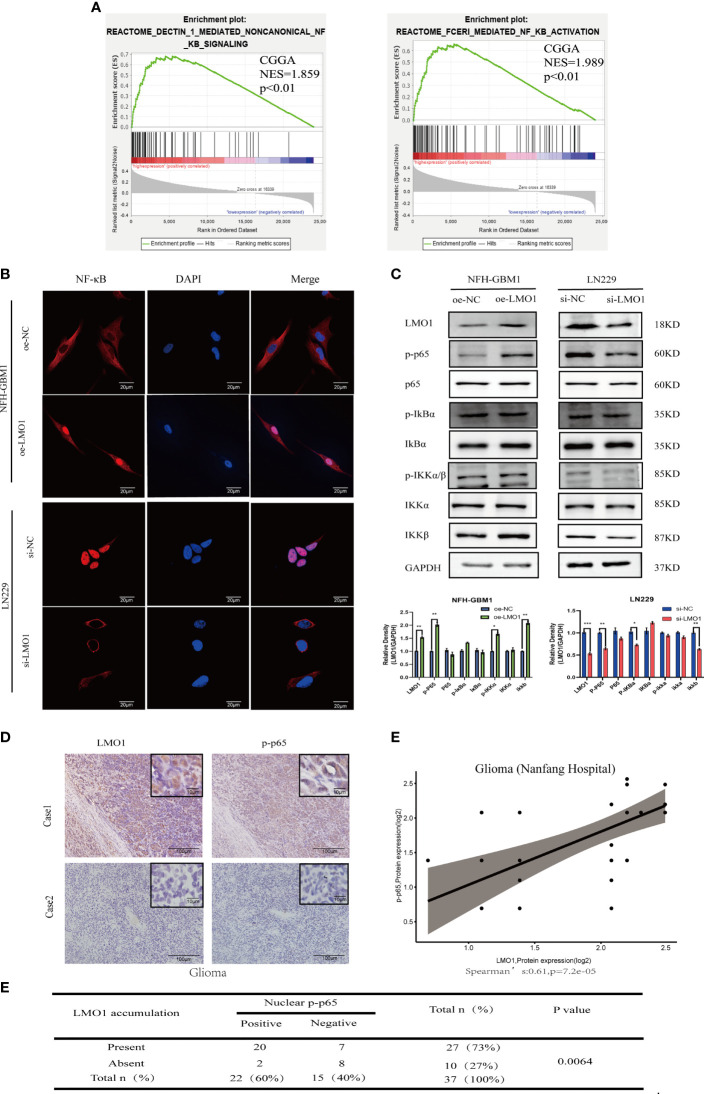
LMO1 regulates the malignant biological behavior of glioma through the NF-κB pathway. **(A)** GSEA analysis showed that high expression of LMO1 was positively correlated with enhanced expression of the NF-κB pathway in CGGA datasets. **(B)** Subcellular localization of NF-κB p65 in the indicated cells as analyzed by an immunofuorescence staining assay.Scale bars=20μm. **(C)** The protein expression of the downstream genes of the NF-κB pathway after LMO1 knockout or overexpression as measured by western blotting and gray quantitative analysis. Three biological replicates were performed. Student’s t test was used for statistical analysis. **(D)** Adjacent tumor sections from representative cases show LMO1 and p-p65 expression in human gliomas. Scale bars=100μm (main images) and 10μm (insets). **(E)** The protein expression of LMO1 and p-p65 is significantly positively correlated in the glioma tissue. (R=0.61, n= 37,p=7.2e-05). **(F)** The relationship between LMO1 and pp65 was analyzed by Spearman rank correlation test (p=0.0064). *p < 0.05. **p < 0.01. ***p < 0.001.

To explore the biological significance of LMO1 in glioma, the knockdown of LMO1 were established in LN229 cells. The empty vector transfected tumor cells served as control groups. The efficiency of LMO1 downregulation was validated with qPCR and Western blot assay (**Figure 3A**). The impact of LMO1 on glioma cells proliferation *in vitro* was then examined. Cell growth was determined by CCK-8 assay within a 6-day monitoring period. Results showed that downregulation of LMO1 could impaired the proliferation of LN229 cells compared with control groups (**Figure 3B**). Inhibition of cell-cell and/or cell-matrix adhesive functions correlated with tumor migration and invasion. However, it was unclear whether LMO1 could affect the migration and invasive ability of glioma cells to influence patient prognosis. The results of scratch assays showed that cell migration inhibited significantly as a result of LMO1 knockdown in LN229 cells (**Figure 3C**). Invasiveness evaluation by transwell invasion assay showed significant difference in LN229 cell between LMO1 knockdown groups and the controls (**Figure 3D**). To further investigate whether LMO1 exerted the above described functions, we generated LMO1 overexpression (OE) in NFH-GBM1 cells that has low level of LMO1 protein. Compared with the empty vector, the enforced expression of LMO1 also promoted cell proliferation, migration and invasion in NFH-GBM1 cells (**Figures 4B–D**). Moreover, western blot analysis verified that the protein expression levels of Vimentin, Slug, Snail and MMP2 decreased in the sh- LMO1 LN229 cells while elevated in the LMO1 overexpression group (**Figure 3A**). These results suggest that LMO1 could promote the proliferation, migration and invasion of glioma cells *in vitro*.

### LMO1 Regulated the NF-κB Signaling Pathway in Human Gliomas

To further explore the possible signaling pathways in which LMO1 regulates the proliferation, migration, and invasion of GBM, we performed GSEA on CGGA dataset. The results showed a significant NF-kB signaling pathway enrichment in the higher LMO1 expression group (**Figure 4A**). Therefore, we examined changes in the NF-κB signaling pathway after LMO1 regulation through immunofluorescence and western blotting. The results showed overexpressing LMO1 significantly increased the NF-κB nuclear level. In contrast, the opposite results were again obtained in the LMO1 knockdown cell lines (**Figures 4B, C**). By ICH analysis, we also found that the accumulation of LMO1 protein is significantly positively correlated with p-p65 expression (n =37; p=0.0064) in human glioma (**Figures 4D–F**). These observations agree with the finding that LMO1 accumulation induces the activation of NF-kB signaling pathway. Taken together, these results demonstrate that LMO1 is an upstream factor modulating the NF-kB pathway in glioma.

### LMO Promotes the Glioma Progression by Regulating NGFR Transcription and NF-κB Activation

**Figure 5:**
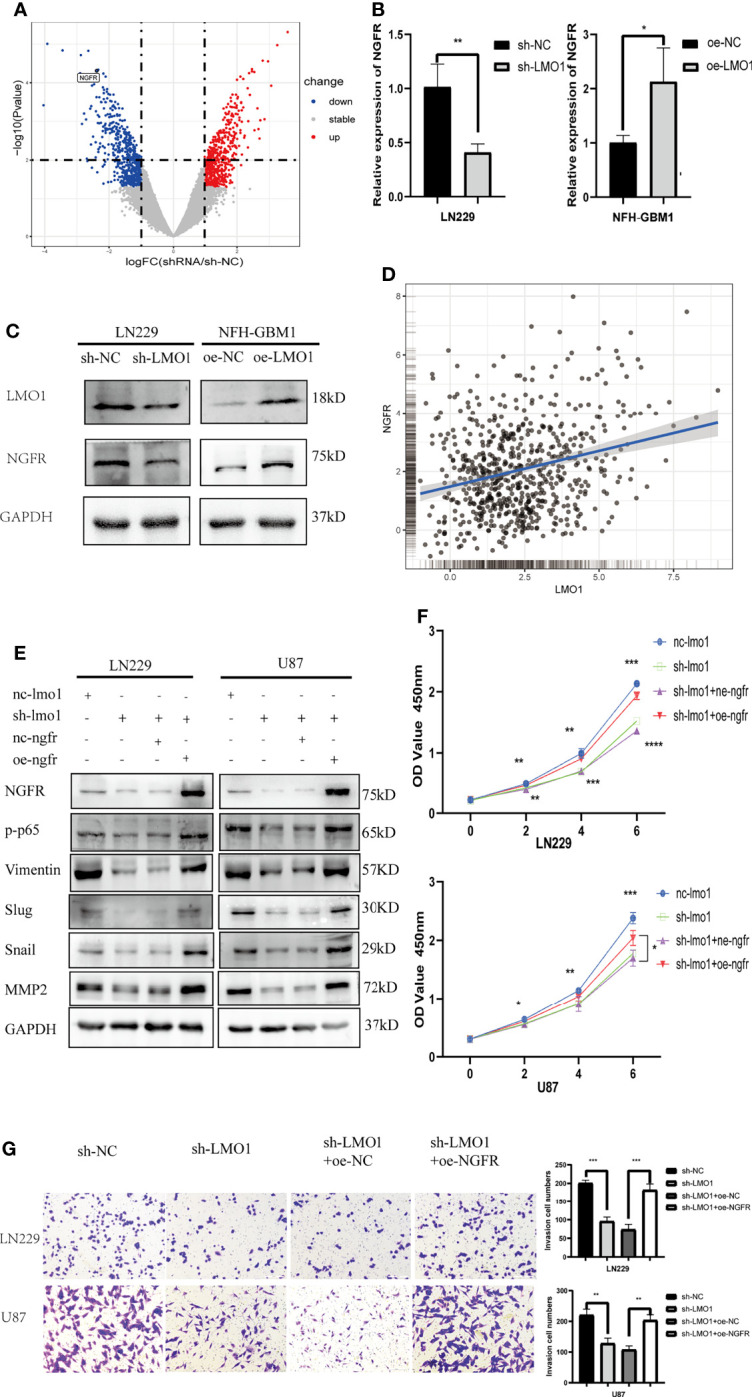
LMO1 promotes the glioma progression by regulating NGFR transcription and NF-κB activation. **(A)** Three independent LMO1 reduced and control cells were prepared for RNA preparation and RNA-Seq analysis. Genes with altered expression were displayed in volcano plots. The upregulated genes are highlighted in red and downregulated genes in blue. Student’s t test was used for statistical analysis. **(B)** LN229 cells were transfected with specific sh-RNA of LMO1 or negative sh-RNA (sh-NC),while NFH-GBM1 cells were transfected with the plasmaid overexpressing LMO1 or empty plasmid. RT-PCR was used to detect the NGFR mRNA level. Student’s t test was used for statistical analysis. **(C)** glioma cells were prepared as **(B)**, western blot was used to detect NGFR protein level. **(D)** Scatter plot of the expression of LMO1 and NGFR in a CGGA dataset. Pearson correlation analysis was used for statistical analysis. (R = 0.26 P < 0.001). **(E)** LN229 and U87 cells were transfected with lentivirus of LMO1 KD or NGFR overexpression plasmid or co-transfected them together. Western blot analysis was used to detect NGFR, p-p65, Vimentin, Slug, Snail and MMP2 protein levels. **(F)** Recovery cck8 assays with the indicated LN229 and U87 cell lines. ANOVA was used to calculate the p-value. **(G)** Recovery Transwell assays with the indicated LN229 and U87 cell lines. Original magnification, 400×. Five random fields of view were captured for each group. Student’s t test was used to calculate the p-value. *p < 0.05. **p < 0.01. ***p < 0.001. ****p < 0.0001.

To gain insight into the mechanism by which LMO1 activate NF-kB pathway, we attempted to perform RNA-seq in LMO1-KD or control cells. We transfected si-RNA into LN229 cells and verified the level of knockdown by WB. RNA-seq analyses were then conducted for LMO1-knockdown/control LN229 cell lines. By statistic analysis, the differential expressed genes were displayed in the volcano plot between the si-RNA and negative control groups(**Figure 5A**). Among those relative downregulated genes, NGFR, which had been confirmed to play a crucial role as a cancer promoter in glioma, was focused on. The LMO1 was knocked down in LN229 cells and overexpressed in NFH-GBM1 cells to confirm the results of the RNA-seq and detect the NGFR expression. Consistent with the result of RNA-seq, the NGFR mRNA and protein expression was upregulated in LMO1 overexpressed cells than control cells by RT-PCR and western blot (**Figures 5B, C**). Subsequently,We also analyze the relationship between LMO1 and NGFR from CGGA database using GlioVis (http://gliovis.bioinfo.cnio.es) (23). A moderate positive correlation existed between the LMO1 expression and NGFR in glioma patients (**Figure 5D**). Several independent data supporting a strong association with NGFR and high-grade glioma have been established by the Repository of Molecular Brain Neoplasia Data (REMBRANDT), the TCGA, and the Human Protein Atlas where NGFR has been shown to be elevated at both the RNA and protein level (24). Furthermore, accompanying patient data establish that high expression of NGFR correlates with lower overall patient survival (24). Taken together, these data revealed that NGFR, which was upregulated by LMO1 in glioma, may induce poor prognosis.

Previous studies revealed that NGF binding to NGFR induces nuclear translocation of p65 and increases NF-kB activity in Schwann and PCNA cells (25). To explore whether activated NGFR is indeed the prime molecular factor that causes the defects in cell proliferation and invasion of LMO1-depleted glioma cells, we performed recovery experiments by transfecting NGFR overexpression plasmid into these LMO1 KD cells and analyze EMT phenotype-related signatures and biological functions. First, Western blot results showed that the levels of p-p65 and NGFR were significantly decreased after LMO1 knockdown in LN229 and U87 cells, exogenous expression of NGFR restored the downregulation of the expression of NGFR, p-p65,Vimentin,Slug,Snail and MMP2 (**Figure 5E**). CCK-8 assay (**Figure 5F**) and Transwell assay (**Figure 5G**) showed that exogenous expression of NGFR also attenuated the suppression of proliferation and invasion caused by shRNA-mediated depletion of the LMO1 protein. These data further demonstrate that LMO1-NGFR-NF-KB axis is essential for driving glioma invasion and progression.

### Downregulation of LMO1 Inhibits Glioma Tumorigenesis *In Vivo*

**Figure 6:**
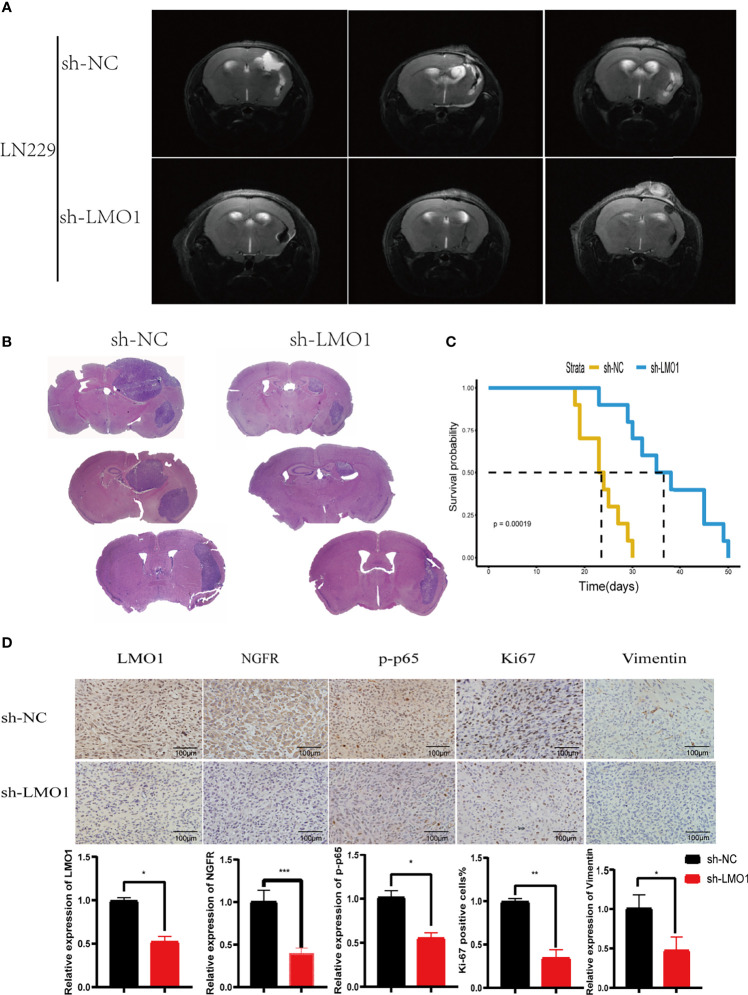
LMO1 silencing inhibits tumorigenesis *in vivo*. **(A)** Cranial MRI T2 sequence images of intracranial tumor-bearing mice in LN229 control or sh-LMO1 cells at 3 weeks after transplantation.**(B)**H&E staining of sections from mouse brains with LN229 control or sh-LMO1 xenografts at 15 days after implantation. **(C)** Survival analysis for animals implanted with LN229 sh-LMO1 or control cells (n control = 6, n sh-LMO1 = 6).Log-rank tests were used to calculate p-values. **(D)** IHC for LMO1, p-p65, Ki-67 and Vimentin in sections from indicated xenografts (scale bar=100μm). the lower panels show histograms of the results. Student’s t test was used for statistical analysis. *p < 0.05. **p < 0.01. ***p < 0.001.

Furthermore, We next explored the biological function of LMO1 using sh-LMO1 lentivirus-infected LN229 cells *in vivo*. MRI scan and Haematoxylin and eosin (HE) staining of intracranial tumour-bearing mice at 3 weeks after implantation suggested that compared with the control conditions, LMO1 knockdown impaired tumor growth (**Figures 6A, B**). Importantly, LMO1 knockdown reduced the progression of xenograft tumor growth and prolonged overall survival in nude mice bearing intracranial tumors (**Figure 6C**). Immunohistochemistry also certified the low expression level of LMO1 and NGFR in sh-LMO1 cells (**Figure 6D**). In addition, a reduction in the proliferation and invasion signature genes (ki67,vimentin) were observed in tumour with downregulated LMO1 (**Figure 6D**). Interestingly, p-p65 expression was significantly decreased in the sh-LMO1 group compared with the control group. These results demonstrated that LMO1 silencing led to reduced proliferation and invasion of glioma cells *in vivo*. Collectively, these *in vivo* and *in vitro* experiments revealed that LMO1 is essential for proliferation and invasion of glioma cells *in vivo*.

### Establishment of Nomogram for Survival Prediction in Human Glioma

**Figure 7:**
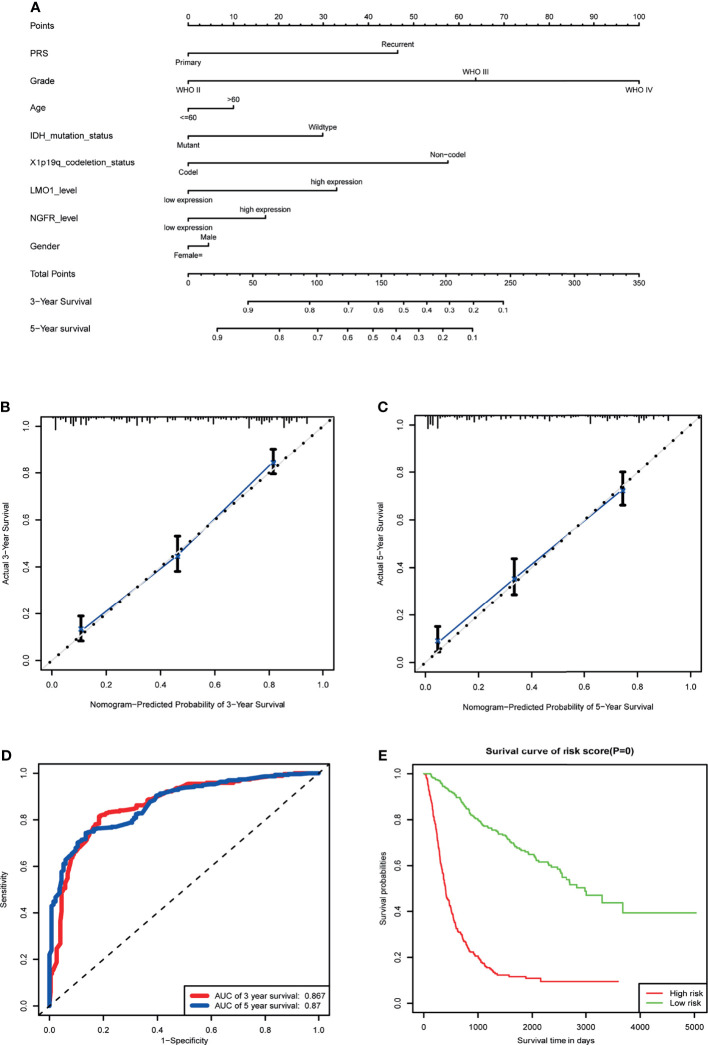
Establishment of the overall survival nomogram for human glioma patients using the CGGA dataset. **(A)** Nomogram for predicting overall survival of human gliomas. There are seven components in this nomogram: PRS, Grade,Age, IDH Mutation Status,1p/19q Codeletion Status, LMO1 and NGFR level, and Gender. Each of them generates points according to the line drawn upward. And the total points of the seven components of an individual patient lie on “Total Points” axis which corresponds to the probability of 3‐year and 5‐year survival rate plotted on the two axes below. **(B, C)** Calibration plots of the nomogram for predicting overall survival rate at 3 year **(B)** and 5 years **(C)**. The predicted and the actual probabilities of overall survival were plotted on the x‐ and y‐axis, respectively. **(D)** ROC curve showing the sensitivity of the program. **(E)** Kaplan‐Meier curves of two risk subgroups stratified by the total points the nomogram gives.

In view of the prognostic value of LMO1 in glioma, we constructed a nomogram and risk classification system for predicting 3- and 5-year survival. In the cohort, 542 glioma cases from the CGGA were included. A Cox proportional hazards model was employed in the cohort to assess the value of each variable in predicting the prognosis of glioma patients. The criteria for selecting variables conformed to clinical relevance and multivariate Cox analysis (26). It has been reported that age, IDH status and sex are associated with the incidence rate or prognosis of glioma (1, 27–29). Considering the clinical factors of glioma, these parameters (LMO1 and NGFR expression level, age, gender, WHO grade, IDH status and 1p/19q co-deletion) were included in the predictive model. The predictive model was presented as a nomogram and is shown in (**Figure 7A**) receiver operating characteristic (ROC) curve was used to evaluate the accuracy of prediction of 3- and 5-year survival in the sets. The area under the curve (AUC) of the nomogram for 3-year survival was 0.867 in the cohort, and the AUC of 5-year survival in the nomogram were 0.87 in the cohort (**Figures 7B, C**). The calibration plot for the probability of survival at 3 or 5 years showed an optimal agreement between the prediction and observation in the cohort(**Figure 7D**).In addition, a risk classification system for predicting the prognosis of glioma patients was developed. Patients in each cohort were divided into low-risk and high-risk groups according to the median cut of value of the risk scores. The Kaplan–Meier curves showed that the high risk group exhibited poorer prognosis than the low-risk group in the cohort (**Figure 7E**). These data suggested that LMO1 is an independent prognostic factor that can be used to competently predict the survival of patients with glioma.

## Discussion

At present, the prognosis of glioma patients is very poor, even when multimodal treatment strategies are used (1, 30). Despite advances in the detection and treatment of gliomas, current therapies remain limited for glioma formation and progression. In this study, we showed that LMO1was highly expressed in gliomas, especially in malignant glioblastoma, and found that the expression of LMO1 increased as the overall survival of patients decreased, which demonstrated that LMO1 plays a significant role in the malignancy of glioma.

LMO1 is a member of a family of transcriptional cofactor genes that encode two zinc-finger LIM domains, forming protein-protein interaction domains (32, 33). Previous studies have suggested that LMO (LIM-only) proteins have essential roles in the central nervous system (CNS) (32, 33). However, despite increasing evidence that this cofactor participate in tumorigenesis and progression of various types of cancers, such as T-cell acute lymphoblastic leukaemia (34), gastric cancer (35), lung cancer (36), and prostate cancer (37), the roles of LMO (LIM-only) proteins in human glioma are unclear. Based on our data, we propose that LMO1 is a novel biomarker of human glioma cells that promote growth and migration by activating NF-kB signaling pathway.

The present study first focused on detecting the expression level of LMO1 in normal brain tissue and tumor tissue obtained from patients with glioma by mRNA level in TCGA, CGGA and GEO dataset. It was observed that expression of LMO1 was significantly higher in high-compared to low-grade gliomas, and may serve as an independent prognostic factor for gliomas. Of note, high expression of LMO1 in the nuclei of tumor cells was observed in great part of the patients with glioma by IHC. Furthermore, the result suggested a upward trend in LMO1 expression with the increase in the degree of malignancy of the tumor, which is consistent to previous results according to which LMO1 expression was widely expressed in human cancers of the lung, gastric, prostate and neuroblastoma.

Enhanced LMO1 expression has also been found to be linked closely with 1p19q co-deletion or MGMT methylation. The impact of LMO1 expression on patients’ survival stratified by these molecular features showed that LMO1 expression could delineate glioma patients together with same other specific genetic alterations. Indeed, these patients could be classified into two subsets with completely distinct clinical outcomes, which were more pronounced than in all patients. LMO1 could facilitate predicting prognosis for patients with IDH wt or 1p/19q co-deletion, suggesting that the prognostic value of LMO1 was dependent on IDH status and 1p/19q co-deletion. In terms of the relationship between LMO1 expression and sensitivity of radiotherapy or chemotherapy, our results showed that LMO1 could contribute to chemoresistance and radioresistance. *In vitro*, our results validate that LMO1 could promote glioma cells proliferation, migration and invasion.

Besides the use of LMO1 as a novel prognostic biomarker of glioma, underlying mechanisms of LMO1 were identified. RNA-Seq and qPCR revealed that LMO1 could be a positive upstream regulator by regulating the mRNA expression of NGFR. NGFR can increase proliferation and invasiveness in in several contexts but has the opposite effect in others. NGFR is also associated with tumorigenesis of melanoma (12), thyroid (38) and breast cancer (39). Functional, biochemical, and clinical studies established that NGFR dramatically enhanced migration and invasion of genetically distinct glioma cells *in vitro* and *in vivo* and frequently exhibited robust expression in highly invasive glioblastoma patient specimens. In lower-grade glioma, however, NGFR has been shown to inhibit tumor growth and survival (40, 41). Our results align with studies in which NGFR acts as a tumor promoter. Knocking down *NGFR* expression decreased cell proliferation rates and the invasive ability of high invasive glioma cells. NGFR KD reduce the protein expression of Epithelial-Mesenchymal Transition(EMT) marker Vimentin,Slug and Snail and the invasive marker MMP2, while overexpression of NGFR *in*creased these protein. EMT has been widely reported as a key mechanism in promoting migration, invasion, and tumor progression in glioma. Moreover, the absence MMP2 protein decreases proliferation and significantly increases survival in mice in a GBM xenograft model. Specially, a significant increase in MMP2 expression corresponding to glioma malignancy grade with the highest peak in glioblastoma. Our phenotypic and molecular data suggest that increasing NGFR expression in glioma cells may promote proliferation and invasion. Our experiment results show that LMO1 may regulate the transcription of NGFR and overexpression of NGFR recover the expression levels of Vimentin,Snail,Snail,MMP2 and p-p65 in LMO1-KD cells. However, our experiments did not fully address the mechanism how LMO1 regulate the transcription of NGFR, which still needs to be further explored. *In vitro* assays suggests that upregulation of the phosphorylation of p65 resulting from NGFR overexpression can drive proliferation and invasion in human glioma cells.

The requirements and characteristics of NF-kB activation *via* NGFR are not completely clear. Previous studies found that the need for a stressful environment for NGFR in order to be able to induce NF-kB nuclear translocation (42). It has been proposed that TRAF-6 can interact with NGFR and is implicated in NF-kB induction in Schwann cells (43). Moreover, there are been several reports that link NGFR signaling to IKBα degradation in other cell types (44). In PC12 cells, selective NGFR activation using a chimeric PDGF/p75 receptor induces NF-kB activation accompanied with an increase in IKK activity (45). Our results indicate that NGFR regulated by LMO1 induce the activation of NF-κB pathway, while silencing LMO1 suppress activation. Because receptors induce different intracellular signal components under environmental and physiological conditions, such as TRAF6, IKBα, and IKK, LMO1’s regulation of NGFR expression may increase the availability of these factors, thereby enhancing the underlying signal.

In this study, we demonstrated that down-regulation of LMO1 inhibits the potential tumorigenicity *in vitro* and *in vivo*. It was confirmed that LMO1 was indeed associated with NF-kB pathway through regulating NGFR. Given that the increase in NGFR levels can significantly elevate the phosphorylation of p65 in LMO1 KD cells, this mean that LMO1 may be a key regulator of glioma proliferation and invasion. However, the exact mechanism of LMO1 regulating NGFR transcription in mediating glioma invasion and progression still needs to be explored.

## Conclusion

In conclusion, our study highlighted that increased LMO1 expression levels were associated with higher tumor grade and poor prognosis in human glioma. A nomogram with LMO1 was constructed and proven to accurately predict 3- or 5-year survival for glioma patients. Regarding biological function, LMO1 facilitated the proliferation, invasion and migration of glioma cells by activation of NF-kB pathway. These findings complement the biological functions of LMO1 and may provide new options for the management of glioma.

## Data Availability Statement

The data supporting the findings of this study are available within the article and its **Supplementary Material**. RNA-seq data and corresponding clinical data for 694 glioma samples were acquired from the TCGA data portal (http://cancergenome.nih.gov/) using the R/Bioconductor package TCGAbiolinks. RNA-seq data and corresponding clinical data for 693 glioma samples were acquired from the CGGA dataset (http://cgga.org.cn/index.jsp). Array expression profiling data and clinical data for 276 glioma samples were acquired from the GEO data portal (https://www.ncbi.nlm.nih.gov/geo/query/acc.cgi?acc=GSE16011).

## Ethics Statement

The studies involving human participants were reviewed and approved by the Ethics Committee of Southern Medical University, China. The patients/participants provided their written informed consent to participate in this study. The animal study was reviewed and approved by The Ethics Committee of Southern Medical University, China.

## Author Contributions

YL and YP designed the research. LG was the major contributor in experiments performing and manuscript writing. JW was the major contributor in experiments performing. HW, YY, ZZ, BN, and XW were contributors in experiments performing and analysis presentation. All authors proofread and approved the final manuscript.

## Funding

The present study was supported by the National Natural Science Foundation (grant nos. 81872442) and President Foundation of Nanfang Hospital, Southern Medical University 2019C007.

## Author Disclaimer

All claims expressed in this article are solely those of the authors and do not necessarily represent those of their affiliated organizations, or those of the publisher, the editors and the reviewers. Any product that may be evaluated in this article or claim that may be made by its manufacturer is not guaranteed or endorsed by the publisher.

## Conflict of Interest

The authors declare that the research was conducted in the absence of any commercial or financial relationships that could be construed as a potential conflict of interest.

## Publisher’s Note

All claims expressed in this article are solely those of the authors and do not necessarily represent those of their affiliated organizations, or those of the publisher, the editors and the reviewers. Any product that may be evaluated in this article, or claim that may be made by its manufacturer, is not guaranteed or endorsed by the publisher.
