# Supplementary material for: LMO1 Plays an Oncogenic Role in Human Glioma Associated With NF-kB Pathway
Source: Front Oncol. 2022 Feb 24;12:770299. doi: 10.3389/fonc.2022.770299 (PMC8907846; doi:10.3389/fonc.2022.770299)
Supplement: Supplementary file 1 [file Table_1.docx]

**Table S1** Information of GBM cell lines

| Cell lines | Organism | Age | Gender | IDH mutation status |
| --- | --- | --- | --- | --- |
| NFH-GBM1 | Homo sapiens | 61 | Male | Wild type |
| NFH-GBM2 | Homo sapiens | 59 | Female | Wild type |
| NFH-GBM3 | Homo sapiens | 49 | male | Wild type |
| LN229 | Homo sapiens | 60 | Female | Wild type |
| U87MG | Homo sapiens | Unknown | Male | Wild type |
| T98G | Homo sapiens | 61 | Male | Wild type |
| U251 | Homo sapiens | Unkown | Unkown | Wild type |
